# Supplementary material for: Are carbon emissions trading and green financial instruments synergistic? -Comprehensive quantitative research based on content analysis
Source: PLoS One. 2024 Mar 7;19(3):e0298601. doi: 10.1371/journal.pone.0298601 (PMC10919601; doi:10.1371/journal.pone.0298601)
Supplement: S1 Data — (ZIP) [file pone.0298601.s001.zip › Data/地级市Z_F工作报告.zip!/║╙─╧/╚2├┼╧┐╩╨/└┤╫╘╣1⁄2╓┌║┼í╛┬φ┐╦╩2╛▌═°í┐_2004.docx]

各位代表：

　　现在，我代表市人民政府向大会作政府工作报告，请予审议，并请各位政协委员和其他列席人士提出意见。

　　一、2003年工作回顾

　　去年，市人民政府在省委、省政府和市委的正确领导下，在市人大、市政协的监督、支持下，团结和带领全市人民，求真务实，开拓创新，圆满完成了市四届人大四次会议确定的各项任务。据初步核算，全市生产总值完成224.14亿元，比上年增长11.6%。

　　大力实施项目带动战略，经济发展后劲明显增强。三门峡火电厂二期、英国开曼公司和东方希望集团氧化铝三大项目开始建设，天元集团再生铝和义马、渑池、陕县4个中型电厂等13个投资上亿元的骨干项目进展顺利，天元集团轿车轮毂一期、黄河铝电电解铝扩建、泉鑫皂素等一批项目相继建成投产。全社会固定资产投资完成76.05亿元，增长29.3%。

　　加快调整工业结构，特色工业的质量和效益明显改善。重点构筑煤电铝及铝制品、黄金、医药、化工、建材五大产业链，天元铝业先进制备基地和义马煤化工基地稳步建设。原煤产量达到1318万吨，发电量76亿度；电解铝生产能力达到22万吨，轿车轮毂100万只；黄金产量47万两；甲醇产量8万吨。国有及非国有规模以上工业增加值完成75.32亿元，增长14.8%。经济效益综合指数132.8%，实现利税27.9亿元，增长12.9%。

　　积极推进农业产业化，特色农业发展势头良好。牧果林三大主导产业产值占农林牧渔业总产值的52.1%，提高2.9个百分点。完成人工造林38.6万亩，建成10万亩苹果、10万亩蔬菜等标准化生产基地。浓缩果汁生产能力跃居全国第二位，畜牧精深加工企业达到18个。灵宝苹果顺利获得国家质检总局“原产地标记注册”，湖滨果汁通过中国绿色食品认证，“三门峡”牌优质烟叶居全省领先地位。

　　努力完善市域城镇体系，城市基础设施建设步伐加快。实施“一城三区”自然山水城市战略规划，快速通道和市区至大坝沿黄公路建成，建设路、和平路和九孔桥改造竣工，青龙涧河大坝蓄水运用，涧河三期治理进展顺利，污水处理厂动工兴建。“双创”和“创优”活动成效显著，荣获“中国优秀旅游城市”称号。义马市区和渑池县城对接规划付诸实施。国道310、209线改造路段通车。

　　进一步优化发展环境，改革开放取得重大突破。深化行政审批制度改革，取消、合并、下放行政审批项目378项，精简52.3%。县级行政服务、国库集中支付两个中心全部规范运行，乡（镇）村财务管理体制改革扎实推进。国有企业改革完成11家，湖滨果汁公司在新加坡成功上市；粮食购销市场化改革全市推开。新增非公有制企业115家，非公有制经济全部入库税金完成6.45亿元，增长12.6%。吸收外商直接投资4199万美元，增长98.1%；出口创汇9523万美元，增长34%。

　　加强财政金融管理，经济形势运行平稳。地方财政一般预算收入完成8.77亿元，增长16.8%。金融机构加大了对地方经济发展的支持力度，各项存款余额增加28.31亿元，贷款余额增加19.54亿元。

　　抗击非典取得重大胜利，公共卫生体系逐步建立。先后投资2300余万元，建立了有效的非典防控体系，确保无疫情输入。市县两级疾病预防控制中心和传染病医院建设全面启动，卢氏农村新型合作医疗制度试点进展顺利。

　　全面发展社会事业，人民生活质量不断提高。实施科技攻关项目94项，科技对国民经济的贡献率有所提高。落实“以县为主”的农村义务教育管理体制，基础教育、职业教育、成人教育协调发展。加强人口与计划生育工作，人口自然增长率4.07‰。国土、森林资源管理力度加大，水、大气污染防治效果明显。开发就业岗位16300个，再就业率49.5%。实现了“两个确保”，对城镇“低保”对象做到了应保尽保。认真落实救灾扶贫工作责任制，重建倒塌房屋9800余间，开仓借粮1450万公斤，稳定解决了4000名农村贫困人口的温饱问题和4.6万人的饮水困难。农民人均纯收入2247元，增长6.3%；城镇居民人均可支配收入6696元，增长7.5%。广播电视和文化体育事业繁荣活跃。实现“全国双拥模范城”二连冠。审计、物价、外事、侨务、对台、民族、宗教、农机、气象、移民、人防、无线电、档案、防震减灾、史志、残疾人等工作也取得了新成绩。

　　社会主义精神文明建设取得新成果，民主法制建设继续加强。在全社会兴起学习贯彻“三个代表”重要思想新高潮，驻村工作收到实效。深入开展“三讲一树”等活动，推动了社会主义精神文明建设。严格执行市人大及其常委会通过的决定、决议，办理人大代表议案、建议96件和政协委员提案227件。坚持依法治市，严厉打击刑事犯罪和经济犯罪活动，被评为全省社会治安综合治理先进市。认真落实党风廉政建设责任制，反腐纠风成效显著。正确处理群众反映的热点、难点问题，确保了社会大局稳定。

　　各位代表，过去的一年，是经济社会发展进程中极不平凡的一年，也是构建区域特色经济体系成效显著的一年。这些成绩的取得，是省委、省政府和市委正确领导的结果，是市人大、市政协大力支持的结果，是全市人民团结奋斗的结果。值此，我代表市人民政府，向工作在全市各条战线上的广大工人、农民、知识分子、干部、人民解放军指战员、武警官兵以及社会各界人士致以崇高的敬意和衷心的感谢！

　　总的来看，去年我们在困难因素增多的情况下取得了令人瞩目的成绩。但也要清醒地认识到，在加快经济社会发展的过程中还存在一些困难和问题：一是农民增收任务艰巨，新出现了一些因灾返贫和需要救助的人口。二是资源型产业比重过大，高技术、高附加值产品少。三是政府债务负担沉重，财政减收增支因素较多，收支矛盾突出。四是国家信贷、出口退税政策和电价调整等因素，将对经济发展产生一定影响。五是经济发展不平衡，有的地方项目拉动不明显。六是一些改革措施不够到位，整顿和规范市场经济秩序的任务依然繁重。同时，个别地方社会治安状况不好，一些干部还存在官僚主义、形式主义的现象。对这些问题，我们必须高度重视，在今后的工作中切实加以解决。

　　二、2004年政府工作的总体要求和预期目标

　　今年是我市实施全面建设小康社会规划的重要一年。从宏观环境看，世界经济和贸易出现增长趋势，两个转移加快，有利于我们扩大对外开放和调整产业结构。从国内形势看，国家采取稳定政策、适度调整的方针，财政转移支付和投资向中西部倾斜，有利于我市资源优势的发挥和优势产业的发展。从我市现状看，近几年新上了一批能源、原材料、加工制造业和基础设施项目，为新一轮经济发展打下了坚实的基础。我们一定要增强加快发展的责任感和紧迫感，抓住机遇，克服困难，谋求大发展，实现大跨越。

　　今年政府工作的总体要求是：以邓小平理论和“三个代表”重要思想为指导，深入贯彻党的十六大和十六届三中全会精神，坚持“发挥比较优势，培育特色经济”的工作方针，围绕“加快发展，维护稳定，狠抓落实”的工作大局，树立科学的发展观和正确的政绩观，按照全面建设小康社会的奋斗目标，加快推进工业化、城镇化和农业现代化，实施投资拉动，推动体制创新，建设服务型政府。正确处理改革发展稳定的关系，维护人民群众的切身利益，加强社会主义政治文明和精神文明建设，促进经济社会全面协调可持续发展。

　　按照上述要求，今年经济和社会发展的主要预期目标是：全市生产总值增长10%；全社会固定资产投资增长20%；社会消费品零售总额增长10%；吸收外商直接投资增长30%，出口创汇增长12.5%；财政一般预算收入增长12%以上；城镇新增就业3万人，城镇登记失业率控制在4.5%以下；农民人均纯收入增长5%以上，城镇居民人均可支配收入增长8%以上；人口自然增长率控制在5‰以内。

　　三、加快推进工业化、城镇化和农业现代化

　　进一步完善区域特色经济体系

　　进一步确立工业在经济社会发展中的核心地位和主导作用，牢固树立“工业兴三门峡兴，工业强财政富，工业发展百姓受益”的意识，坚持把工业发展作为经济工作的重中之重，走“工业富市”之路，以加快推进工业化为龙头，带动城镇化，促进农业现代化，统筹城乡经济协调发展。

　　（一）大力推进工业化，实现工业经济发展的新跨越。继续实施项目带动战略，强力推进项目建设。三门峡火电厂二期、英国开曼公司和东方希望集团氧化铝三大项目，以及天成公司4万吨烧碱、韶星20万吨氢氧化铝等工程要确保完成投资计划。天元集团轿车轮毂二期和钎焊铝箔、灵宝绿源20万吨果品综合加工、义马2×20万吨甲醇一期、金渠和灵宝两个黄金探矿等项目要尽快开工。天元集团再生铝、亚能天元2＃机组、义马锦江电厂、惠能热电厂一期、渑池生物制药等项目今年要建成投产。华能义马电厂4×60万千瓦机组一期、金渠集团2亿克拉人造金刚石等重大项目前期工作要加快推进，切实抓好新签约的三化集团60万吨甲醇、渑池2万吨铝型材、灵宝林板纸一体化等项目的跟踪落实工作。同时，要进一步搞好项目储备，保证产业接续。

　　围绕建设“五大基地”，壮大支柱产业。能源基地，要抓好7个电厂和1个煤矿建设，力争今年新增电力装机容量45万千瓦；煤化工基地，要集中抓好总投资19亿元、13个规划项目的实施进度；铝工业基地，要抓好氧化铝、轿车轮毂和复合铝箔等项目建设，今年要达到5万吨再生铝、200万只轿车轮毂生产能力；黄金生产加工基地，重点抓好深部探矿和市外采矿，引进新工艺，搞好精深加工；林果业生产加工基地，突出抓好果品加工和林板、林纸一体化，扩大产业规模。

　　加快改造传统制造业，培育高新技术产业。引进先进技术，提升精密量仪、水工机械、化工成套设备、机床、轴承等传统制造业水平，增强市场竞争力。加强与高等院校和科研院所的合作，重点抓好“863计划”泡沫铝等高科技项目的实施，支持电解铜箔、泉鑫双烯、高科技新药等45种高新技术产品生产，推动高新技术产业发展。

　　壮大企业集团，提高工业运行质量。把部省属企业纳入构建区域特色经济体系发展规划，支持矿产资源向优势企业聚集。积极促进企业联合和重组，引导中小企业围绕大企业的主导产品搞好配套生产，尽快形成一批以能源、煤化工、铝工业、黄金为主的大型企业集团。搞好工业运行的监测和预警，加强资金和资源的调度，促使企业开足马力生产。完善企业技术创新和激励约束机制，提高经营效益，增强工业经济实力。

　　加快投融资体制改革步伐，积极筹措建设资金。加强政府投资管理，整合投资资源，促进建设资金的优化配置。抓紧建立“谁投资、谁受益，谁决策、谁承担风险”的新型投资机制，取消对基础设施投资主体的一切限制，实现投资主体多元化。积极探索政府投资退出机制，推动已建成的项目依法转让产权或经营权。激励引导金融机构加大对地方经济发展的支持力度，重点组织银企合作洽谈等活动，以满足企业经营发展的资金需求。加大拟上市公司的培育和推荐力度，推动优质企业上市。

　　（二）坚持用工业的理念发展特色农业，推进农业现代化。把解决“三农”问题作为农村工作的重点，全面落实促进农民增收的政策措施。坚持“多予、少取、放活”的方针，在去年取消一般农业特产税的基础上，今年农业税再降3个百分点，直接减负2000余万元。要采取措施，确保对农民的粮食直接补贴到位、农资价格管理到位、农业税减负核算到位。巩固农村税费改革成果，防止农民负担反弹。加大财政对农业的支持力度，支农资金的增长要高于财政收入的增长幅度。积极运用支农再贷款，支持农村信用社继续加大对农村经济发展的信贷扶持。

　　加快农业产业化步伐，扶大做强农业龙头企业。突出抓好牧果林等支柱产业发展，集中建设肉牛、奶品、牧草、苹果、速生丰产林、蔬菜、烟叶、食用菌等优质农产品基地。实施名牌战略，争取把湖滨果汁等已有的省名牌培育成中国名牌，重点推出SOD苹果等新品牌，积极做好原产地域产品保护工作。依托现有农副产品深加工企业和大型农贸市场，拉长产业链，扶持壮大果汁、板材、肉奶制品等农业龙头企业，推进农业现代化。

　　积极推进农业标准化，提高农产品质量和效益。按照国际质量安全标准，实施优质农产品区域化布局、标准化生产，把农业生产的产前、产中、产后等环节纳入标准化管理。全面推行农产品质量检测，提升特色农产品质量和市场竞争力。切实加强农业信息化网络等服务体系建设，努力开拓国内、国际两大市场。

　　调整优化农村经济结构，发展壮大县域经济。大力发展农村二、三产业，围绕特色农产品生产，搞好农副产品深加工，加快发展农村工业。抓好城乡集贸市场建设，搞活农副产品流通。把县域经济作为构建区域特色经济体系的基石，坚定不移地改组改造国有企业，各县（市）区每年都要上3—5个较大的工业项目，扩大县域工业规模，促进农工贸一体化，加速产业聚集，形成“板块经济”。

　　加强基础设施建设，改善农村生产生活条件。抓好槐扒提水工程西段村水库、豫西农业综合开发等续建项目，开工建设气象雷达站、窄口灌区改造等大型工程，发展节水灌溉6000亩，治理水土流失80平方公里，解决5万人和2万头大牲畜饮水困难。加快卫家磨水库复建、氟改水工程等项目的前期工作，争取早日开工。抓好省道322线卢氏至栾川界改建工程，完成新改建县乡公路164公里。

　　（三）扎实推进区域中心城市发展，提高城镇化水平。高标准、高质量完善市域城镇体系规划。按照构建以三门峡市区为区域中心，以义马市区和渑池县城、灵宝市区为两翼，以310国道为轴心，东西呼应、带状发展城镇带的要求，对初步规划方案广泛征求意见，组织专家论证，与实施UNDP项目相结合，抓紧完成“一城三区”中心城市总体规划，及时安排搞好详规。同时，要完成副中心城市、重点镇的分级规划。

　　重点抓好区域中心城市的建设和管理。东城区建设要以提高人民生活质量为目的，加快完善城市功能。涧河治理在搞好三期扫尾的基础上向东推进，快速通道二期、209国道市区段改造和大岭路、经一路改造抓紧施工，上阳大桥建设加快进度，争取今年全部完工。污水处理厂年内投入使用，积极实施城市集中供热。西城区建设要严格实施规划控制，统筹安排交通、通信等基础设施，工业布局有计划地向西区转移，建成产业集中度高、规模优势突出的工业园区。强化城市经营和管理，全面实行城市经营性国有土地收购储备和国有经营性土地使用权招拍挂公开出让制度，强力推行城市自来水、道路保洁、污水处理等公用事业市场化改革。加大城市管理力度，坚持建设与管理并重，建立完善长效机制。全面放开户籍限制，逐步实行一元化管理，吸引农村人口向城市转移，增强中心城市的辐射带动力。

　　全力争创国家园林城市。加快中部自然生态区建设步伐。苍龙涧河大坝年底建成，实现市区常年水面5000亩；白天鹅观赏区建设要完成区内绿化、道路硬化等基础工作。大力实施城郊黄土丘陵沟壑生态治理工程，把环绕市区南山北岭绿化作为一项重点工程来完成。改革义务植树制度，实行社会筹资与群众捐助相结合，通过市场机制、公司运作的模式，提高绿化成效。全面开展庭院绿化活动，掀起创建园林单位高潮，争取三年建成国家园林城市。

　　快速推进副中心城市和城镇带建设。东部义马市区和渑池县城要按照统一规划，进一步搞好建设对接，大力推进灵宝涧北新区建设和卢氏新区开发。加强对省定重点镇建设的指导，突出地方特色，培育支撑产业，加强生态保护，完善公共服务体系。积极配合国家做好郑（州）西（安）铁路客运专线的前期工作，重点推进交通等重大基础设施项目。加快实施城镇带规划，建设“工业走廊”，逐步形成经济发展的隆起带。

　　四、实施“四管齐下”

　　增强区域经济综合竞争力

　　加快机制与体制创新，突出抓好国有企业改革、发展非公有制经济、扩大对外开放、建设服务型政府，进一步优化环境，增强经济发展的动力和活力。

　　（一）深化国有企业改革。要以产权制度改革为重点，积极吸引非公有资本参与国有企业改革，加快股份制改造步伐。坚持分类指导，因企制宜，一企一策、一企多策，全市上半年要完成30家国有企业改革，年底基本结束。按照建立现代企业制度的要求，完善公司法人治理结构。加快分离企业办社会职能，市属企业办学校要尽快完成分离，鼓励引入各种成份，参与企业医院重组和物业管理。坚持公开、公平、公正的原则，实行市场化运作，规范企业改制。同时，要积极推进流通、城建、交通等系统企业的改革，提高经营效益。

　　（二）大力发展非公有制经济。要彻底解放思想，不折不扣地落实促进非公有制经济发展的一系列优惠政策，搞好优质服务，努力营造亲商、安商、惠商的浓厚氛围。凡是国家法律、法规没有明令禁止的行业，都要向非公有制经济开放。积极鼓励非公有制经济参与城市公用事业、房地产、交通、能源、水利、林业、环保等基础设施的建设与经营。按照把旅游业培育成新兴支柱产业的要求，深度开发旅游资源，精心包装重点旅游产品，推向国内外市场。大力发展以连锁经营为主的现代流通业，支持社会力量兴办教育、卫生、体育产业，大力发展信息、中介服务等新兴服务业，促进第三产业发展。

　　（三）全方位扩大对外开放。认真落实省、市发展外向型经济的政策措施，严格兑现奖惩。创新招商引资方式，充分发挥企业在招商引资中的主体作用，更好地依托优势资源、优势产业、重点项目、城镇带建设等招商引资平台，优选招商项目，以商招商、以外引外，加强跟踪、服务和协调，使外商引得来、留得住、能赚钱。支持更多的企业取得外贸经营权，落实国家出口退税政策，巩固扩大铝及铝制品、浓缩果汁、刚玉砂等大宗商品出口规模，积极组织机电产品、农产品和高新技术产品出口。强力实施东引西进，吸引沿海发达地区产业向我市转移。鼓励机械加工、矿山开采、林果业生产加工等优势企业，开拓国内外市场。大力发展劳务经济，有计划组织劳务输出，今年力争完成10万人。

　　（四）努力建设服务型政府。继续深化行政审批制度改革，认真执行《行政许可法》，清理不符合行政许可的规定，清理实施行政许可的主体。坚持和完善反腐败领导体制和工作机制，认真抓好部门行风建设，切实从源头上预防和治理腐败，切实解决损害群众利益的突出问题，营造清廉高效的服务环境。坚持实事求是，把党的路线、方针、政策与当地实际紧密结合，用足用活上级出台的扶持措施，保持政策的连续性，营造宽松开明的政策环境。认真落实行政执法责任制、过错追究制和评议考核制，加强执法队伍建设，营造公正严明的法治环境。全面推行承诺制度，实行政务公开，建设诚信政府；严厉打击制假售假等违法犯罪活动，整顿和规范市场经济秩序；强化全民信用意识，健全中小企业信用评审制度，营造诚实守信的信用环境。

　　五、大力发展各项社会事业

　　 进一步提高人民生活质量

　　坚持实施可持续发展、科教兴市和人才强市三大战略，统筹发展其他各项社会事业，努力提高人民生活水平。

　　（一）全面实施“三大战略”。牢固树立全面协调可持续的发展观。认真落实人口与计划生育目标管理责任制，提高出生人口素质。健全资源有偿使用机制，优化资源配置，依法有效利用、节约和保护水、土地、矿产、森林等资源。继续抓好天然林保护、退耕还林、自然保护区生态工程建设，加快风景名胜区和城乡绿化。以水污染防治为重点，加强市域黄河沿线和南水北调中线工程水源地等重点流域的污染治理。严格执行建设项目环境影响评价和“三同时”制度。对现有企业不能稳定达标的，实施限期治理；凡污染严重、达标无望的企业，坚决予以关停或转产。将不符合城市功能区划要求的企业尽快搬出城区，提高城市环境质量。

　　坚持科教兴市。把教育摆在优先发展的战略地位。进一步完善“以县为主”的农村义务教育管理体制，落实“三保”政策，改善农村办学条件，巩固提高“两基”水平。加快教师队伍建设，全面推进素质教育。鼓励和扶持民办教育，重视发展职业技术教育，有针对性地开展农民实用技术培训。深入开展科技攻关和科技兴农活动，提高科技创新能力。大力发展民营科技企业和科技中介机构，促进科技成果加速转化为现实生产力。

　　实施人才强市。创新人才工作机制，抓好人才开发，充分发挥现有人才的作用，最大限度地营造干事创业的宽松环境；进一步优化人才结构，积极引进经济社会发展的急需人才，落实优惠政策，力求引得来、留得住、用得好。深化人事制度改革，全面推行考试录用、竞争上岗和事业单位聘任制。

　　（二）加强公共财政体系建设。依法强化税收征管，加强非税收入管理，确保财政收入稳定增长。积极争取更多的财政转移支付资金，增加可用财力。继续坚持“一是吃饭，二是还账，三是建设”的原则，确保工资发放，增加国有企业改革、扩大就业的支出。加强政府债务管理和监督，按照分级负责的原则，有计划安排偿债资金。进一步深化部门预算，推行国库集中收付和政府采购制度，确保财政资金运行规范、安全。

　　（三）推进各项社会事业不断进步。完善公共卫生体系，改建市传染病医院，加快市疾病控制中心和6个乡（镇）中心卫生院建设，进一步抓好农村新型合作医疗制度试点工作。繁荣文学艺术、广播电视和新闻出版事业，加强社区文化、企业文化和村镇文化建设，重视文物保护和利用。广泛开展全民健身活动，努力提高竞技运动水平。健全经济运行监测体系，组织好第一次经济普查。加强国防教育，关心、支持驻军和民兵预备役建设，深入开展拥军优属和军民共建活动，争取“全国双拥模范城”三连冠。继续做好审计、外事、侨务、对台、民族、宗教、人防、防震减灾、无线电、档案、史志等工作。

　　（四）努力提高人民生活水平。落实小额贷款、税费减免等优惠政策，鼓励下岗失业人员自主创业、自谋职业。重视发展劳动密集型产业，积极推进城镇社区服务，开发就业岗位。加强职业介绍和再就业培训，多渠道做好大中专毕业生就业和复员转业军人安置工作。认真清理整顿建设领域拖欠工程款和农民工工资问题，为农民工进城务工创造平等的就业机会。

　　高度重视社会保障体系建设。落实社会保障政策，确保下岗职工基本生活费和企业离退休人员养老金按时足额发放，确保城镇“低保”对象应保尽保，努力做好老龄工作。积极稳妥地推进下岗职工出中心与失业保险并轨，加大城镇个体、私营和三资企业社会保险费征缴力度，依法把应参保企业和职工逐步纳入社会保险范围。切实搞好扶贫开发工作，落实科技扶贫、搬迁扶贫措施，大力实施扶贫项目。完善困难救助制度，搞好生产救灾，确保实现救灾工作目标。

　　（五）加强社会主义精神文明建设和社会治安综合治理。认真学习党的十六大、十六届三中全会精神和“三个代表”重要思想，努力形成学习型社会。全面落实《公民道德建设实施纲要》，继续开展“道德规范进万家、诚实守信万人行”和“双创”等群众性创建活动，加强社会公德、职业道德和家庭美德教育。积极组织科技、文化、卫生“三下乡”活动，倡导科学文明健康的生活方式，提高全民文明素质。

　　贯彻“严打”方针，依法打击严重刑事犯罪、带黑社会性质的团伙犯罪和流氓恶势力，惩治经济犯罪。巩固社会治安综合治理成果，加强防控体系建设。严密防范境外敌对势力、恐怖分子的渗透破坏活动，坚决同“法轮功”等邪教组织作斗争。强化安全生产责任制，坚决遏制重特大事故的发生。高度重视人民来信来访工作，做到有访必接、有案必查、有查必果。

　　六、进一步转变作风

　　加强政府自身建设

　　各级政府和广大公务员必须认清在全面建设小康社会进程中所担负的艰巨任务，主动适应新形势，高度重视自身建设，不断提高行政能力和管理水平。

　　（一）坚持科学的发展观和正确的政绩观，大兴求真务实之风。坚持以人为本，办事情、作决策都要把为人民群众谋利益作为根本目的，坚持一切从实际出发，积极进取，量力而行，不盲目攀比；坚持办实事，求实效，珍惜民力，不搞劳民伤财的“形象工程”；坚持统筹兼顾，立足当前，着眼长远，不急功近利。要强化大局意识和责任意识，真正沉下心思、集中精力、真抓实干，做到为官一任、造福一方、惠及后代。

　　（二）转变政府职能，提高决策水平。抓紧完成市县政府机构改革，严格控制机构、编制和人员。积极转变政府职能，切实把政府的经济管理职能转变到为市场主体服务和创造良好发展环境上来，把政府的社会管理职能转变到提供良好的公共服务和更多的公共产品上来。加快建立健全各种突发事件应急机制，提高政府应对公共危机的能力。建立完善重大问题集体决策制度、专家咨询制度、社会公示和听证制度，保证决策的科学性和正确性。

　　（三）全面推进依法行政，自觉接受人民监督。各级政府及工作人员要严格遵守宪法和法律，坚决执行同级人大及其常委会的决议、决定，主动接受人大的法律监督和政协的民主监督，认真办理人大代表建议和政协委员提案。进一步加强与各民主党派、工商联和无党派人士的联系，充分发挥工会、共青团、妇联等人民团体的桥梁纽带作用。健全基层自治组织和民主管理制度，扩大基层民主。

　　（四）加强公务员队伍建设，树立政府良好形象。政府公务员要牢固树立执政为民的思想，牢记全心全意为人民服务的宗旨，坚持解放思想、与时俱进、大胆创新，做到忠于职守、公道正派、乐于奉献。各级政府要从严治政，严格考核，努力建设一支政治坚定、业务精通、清正廉洁、作风优良的公务员队伍，全面提高政府的管理水平和工作效率。

　　各位代表，机遇和挑战并存，希望与困难同在。让我们在以胡锦涛同志为总书记的党中央领导下，高举邓小平理论伟大旗帜，全面贯彻“三个代表”重要思想，抢抓机遇，扎实苦干，加快发展，奋勇争先，为做中原崛起“第一梯队”、提前实现全面建设小康社会的宏伟目标而努力奋斗!
